# Artificial Intelligence: A Shifting Paradigm in Cardio-Cerebrovascular Medicine

**DOI:** 10.3390/jcm10235710

**Published:** 2021-12-06

**Authors:** Vida Abedi, Seyed-Mostafa Razavi, Ayesha Khan, Venkatesh Avula, Aparna Tompe, Asma Poursoroush, Alireza Vafaei Sadr, Jiang Li, Ramin Zand

**Affiliations:** 1 Department of Public Health Sciences, College of Medicine, The Pennsylvania State University, Hershey, PA 17033, USA; vidaabedi@gmail.com; 2 Prime Healthcare, Saint Mary’s Regional Medical Center, Reno, NV 89503, USA; razavi.mos@gmail.com; 3 Geisinger Neuroscience Institute, Geisinger Health System, Danville, PA 17822, USA; akhan2@geisinger.edu; 4 Geisinger Health System, Geisinger Northeast Internal Medicine Residency, Wilkes Barre, PA 18711, USA; 5 Department of Molecular and Functional Genomics, Geisinger Health System, Danville, PA 17822, USA; vavula1@geisinger.edu (V.A.); jli@geisinger.edu (J.L.); 6 Geisinger Health System, Transitional Year Residency Program, Bloomsburg, PA 17815, USA; aparnatompe@gmail.com; 7 Department of Biomedical Engineering, University of Memphis, Memphis, TN 38152, USA; prsroush@memphis.edu; 8 Department of Biomedical Engineering, University of Tennessee Health Science Center, Memphis, TN 38163, USA; 9 Départment de Physique Théorique and Center for Astroparticle Physics, University of Geneva, CH-1211 Geneva, Switzerland; vafaei.sadr@gmail.com

**Keywords:** healthcare, artificial intelligence, cerebrovascular diseases, cardiovascular diseases, cardio-cerebrovascular diseases, machine learning

## Abstract

The future of healthcare is an organic blend of technology, innovation, and human connection. As artificial intelligence (AI) is gradually becoming a go-to technology in healthcare to improve efficiency and outcomes, we must understand our limitations. We should realize that our goal is not only to provide faster and more efficient care, but also to deliver an integrated solution to ensure that the care is fair and not biased to a group of sub-population. In this context, the field of cardio-cerebrovascular diseases, which encompasses a wide range of conditions—from heart failure to stroke—has made some advances to provide assistive tools to care providers. This article aimed to provide an overall thematic review of recent development focusing on various AI applications in cardio-cerebrovascular diseases to identify gaps and potential areas of improvement. If well designed, technological engines have the potential to improve healthcare access and equitability while reducing overall costs, diagnostic errors, and disparity in a system that affects patients and providers and strives for efficiency.

## 1. Introduction

Artificial intelligence (AI) focuses on how computers learn from large and complex datasets by mimicking the human thought process. AI has the potential to accelerate the field of precision medicine by helping practitioners to calculate the risk, guide the treatment, predict the outcome, and close the care gap using scalable computational resources and advanced algorithms applied to a growing body of data and knowledge. AI can be specifically designed to improve clinical care and increase efficiency in drug discovery [1]. Carefully designed and implemented electronic health record (EHR)-AI embedded tools and applications can save valuable time and assist practitioners with critical decision-making at the point of care. AI can potentially improve health disparity and address implicit bias. Machine learning (ML), an application of AI, provides systems with the ability to learn from data and experiences [2]. 

**Figure 1:**
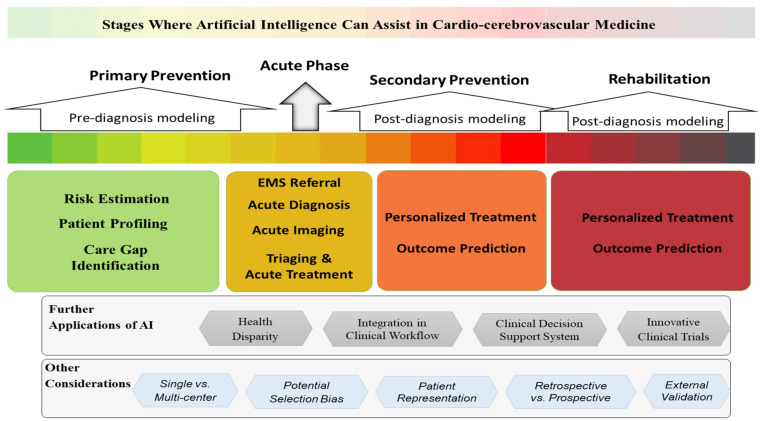
Stages of the care management where artificial intelligence (AI) can add value in cardio and cerebrovascular fields.

Cardio-cerebrovascular diseases, a leading cause of mortality and disability in the United States and worldwide [3,4], have been targeted by big data science and AI applications. Furthermore, with growing vascular risk factors, trends in mortality and complications will be increasing [5]. Many large studies in cardiovascular medicine use AI to provide a promising set of assistive tools to cardiologists and push the boundaries of translational science. Cardiovascular and cerebrovascular diseases share many predictors, pathophysiology processes, among others [6,7,8]. However, big data and advanced prediction modeling have not been studied in the same way in the cardio and cerebrovascular fields. Our intent in this work was to perform a review of the recent AI-enabled applications developed for cardiovascular and cerebrovascular conditions for different stages of care management (Figure 1).

## 2. Methods

**Figure 2:**
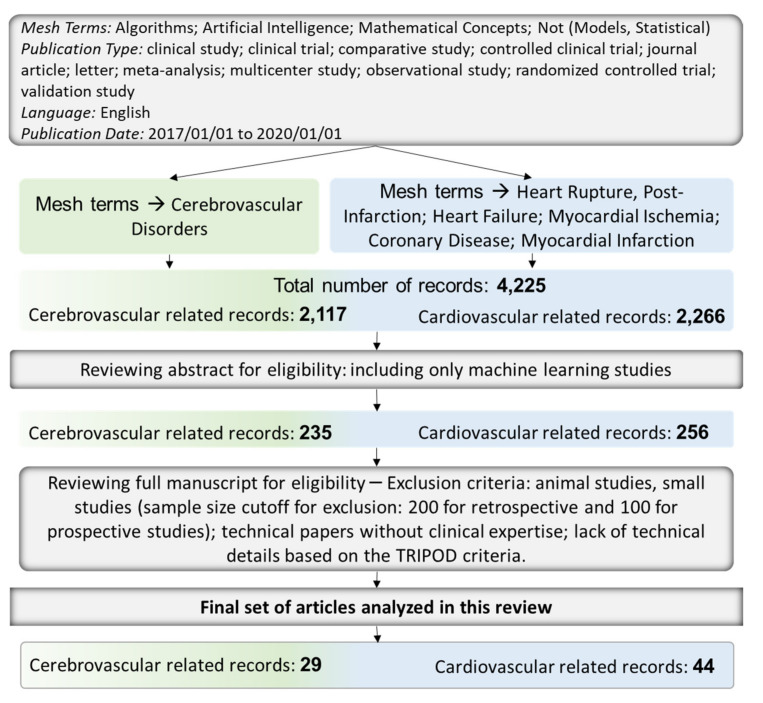
Flowchart for inclusion of studies in the review article.

We conducted a comprehensive literature search to extract original contributions in the various areas of AI application in cardio-cerebrovascular diseases published between 2017–2020. We defined cardiovascular diseases as ischemic heart disease, heart failure, myocardial infarction, and hypertrophic diseases, excluding arrhythmias, infiltrative cardiomyopathies, and genomics. Cerebrovascular diseases were defined as stroke (hemorrhagic/ischemic), thrombosis, and cerebral aneurysmal disorders, excluding genomics. The detailed search criterion is outlined in Figure 2. We examined 256 articles in the field of cardiovascular medicine and included 44 studies in this review article. Similarly, we reviewed 235 studies in cerebrovascular diseases and included 29 studies in this review. We assessed the reporting quality of the studies based on the TRIPOD (transparent reporting of a multivariable prediction model for individual prognosis or diagnosis) statement for including studies in this review [9]. We further divided the studies based on the clinical application; pre-diagnostic, diagnostic/ imaging, and post-diagnostic. Other developing areas of AI research, such as AI in clinical trials and subtyping, AI-powered clinical decision support systems, as well as application of AI in reducing health disparity and implicit bias, have also been briefly discussed.

## 3. Results

A total of 73 cardio-cerebrovascular studies were identified and included in this review. More specifically, 29 studies were cerebrovascular, while 44 studies included cardiovascular diseases (Table 1 and Table 2), with the majority of the cerebrovascular study designs being single-center and retrospective. The reviewed studies were divided into the following categories: Risk stratification modeling (11 cardiovascular, 5 cerebrovascular), Diagnostic studies (4 cardiovascular, 5 cerebrovascular), Outcome prediction and prognosis (18 cardiovascular, 6 cerebrovascular), Treatment strategies (3 cardiovascular, 2 cerebrovascular), and Diagnostic imaging studies (8 cardiovascular, 10 cerebrovascular). Table 1 and Table 2 provide a detailed description of the included studies categorized as mentioned above. The text that follows will further subcategorize the studies to better dissect the various fields of application of AI. The pertinent subsections are also mentioned in the tables to improve readability. 

### 3.1. Application of AI in Pre-Diagnosis Modeling: Primary Prevention

(a) Risk Estimation

Risk assessment tools are becoming more salient in the era of precision medicine. EHR and administrative databases in conjunction with advanced applications of AI have been the driving force behind primary prevention strategies for cardiovascular and related conditions (Table 1). Some of the noteworthy applications using ML for risk estimation included an improved prediction of cardiovascular risk factors in patients with no prior risk factors [10], prediction models of long-term risk of MI and cardiac death in asymptomatic patients [11], and using ML to identify cardiovascular disease risk factors in patients with no initial indications [12,13]. Researchers also looked at the association of biomarkers such as hemoglobin A1c (HbA1c) and thyroid-stimulating hormones, and the use of machine learning (support vector machine, SVM) to identify participants who later developed coronary heart disease [14]. Another study utilized AI-enabled tools in imaging to evaluate the prediction of major cardiovascular events in asymptomatic patients [15]. Predicting survival via ML utilizing echocardiography and CT angiogram (CTA) has also been attempted with promising results [16,17]. Four large-scale studies, mainly from Asian countries, have focused on estimating the risk of cerebrovascular disease (Table 2) [18,19,20,21]. These studies have sought to estimate the risk of stroke in patients with atrial fibrillation. Cerebrovascular studies on risk stratification are mostly retrospective and suffer from limited diversity and smaller sample sizes compared to cardiovascular studies. For instance, in some cardiovascular studies, existing clinical trials have been leveraged (MESA cohort [22] and EISNER trial [23]) with rich extended longitudinal follow-up data (up to ten years); cerebrovascular studies, on the other hand, have a relatively narrower timeline (up to two years).

(b) Clustering and Patient Profiling Before Event

Researchers have used ML to group cardiovascular patients based on coronary artery disease (CAD) severity [24], ischemia scoring [25], obstructive disease [26], and coronary stenosis [27]. ML has also been used to discriminate between healthy individuals and patients with impaired functional reserve due to heart failure with preserved ejection fraction (HFpEF) [28]. With regard to cerebrovascular disease, investigators have implemented ML to improve aneurysm detection with time-of-flight MR angiography [29]. Patient clustering has numerous potential benefits for the patients and the health system. Besides cardiovascular and cerebrovascular diseases, patient profiling has been valuable in other complex diseases [30,31,32,33,34].

(c) Care Gap Identification and Personalized Prevention

Identification of care gaps in medical management is an important potential field for ML with high clinical value. This field is not fully developed in either cardio or cerebrovascular diseases and can be a potential new venue for exploration and advanced application of AI for improving the quality of care and resource optimization. 

In the period of studies collected for this article, only four studies were identified to concentrate on minimizing the healthcare gap. On the cardiovascular front, ML has been used to develop a risk calculator to aid with the initiation of statin therapy for CAD, which can potentially minimize future cardiovascular events in the affected patients [13]. By reclassifying CTA results, ML has been successful in better predicting existing ischemia and distinguishing that from subclinical coronary stenosis [27]. One cerebrovascular study to use ML for closing the care gap focused on better detection of cerebral aneurysms in MR angiography image data [29]. Karlsson et al. assessed an ML-powered clinical decision support system (CDSS) for stroke prevention in a randomized clinical trial on patients with atrial fibrillation (AF). The study corroborated that the CDSS can increase guideline adherence for anticoagulation therapy among these patients [35]. 

Personalized prevention is another area with potential clinical value. Thus far, ML has only been utilized to predict obstructive coronary disease on myocardial perfusion imaging as a directive for preventive action at an individual level [26].

### 3.2. Application of Computational Algorithms in Diagnosis and Acute Phase Treatment

(a) Emergency Medical Services (EMS) Proper Referral

Quality of recovery, in both MI and stroke patients, is dependent on the time from symptoms to intervention [36,37,38]. AI can aid in shortening this time window and improving treatment outcomes. However, there are technological barriers, including access to real-time patient data for model prediction, that make this space complex in terms of its implementation. For instance, in a study by Potter and colleagues, computational algorithms were used for developing an AI-aided system to more promptly identify and refer STEMI patients for cardiac catheterization during the EMS encounter [39]. Using this method for "physician-less" cardiac catheterization lab activation was safe and effective in improving treatment delay with sustainable results over time. To this end, investment in this emerging application of AI can help save lives while reducing systemwide cost and physician burnout due to stress that is due to the patient’s higher risk for disability and death.

(b) Acute Diagnosis

ML can be an essential tool to guide physicians in the acute diagnosis of cardio- and cerebrovascular disease. Most ECG recording devices now possess computational abilities to calculate measurements and “read” ECGs in real-time with variable accuracy [40]. With recent advances in computational algorithms, ML has been used to develop advanced diagnostic systems that can make predictions and direct the pre-hospital diagnosis of acute coronary syndrome [39,41].

Timely diagnosis of ischemic and hemorrhagic stroke, while challenging for physicians, is invaluable for the patient. ML has been explored by researchers for stroke screening [42], detection of stroke and large vessel occlusion using CTA imaging [43,44], detection and subtyping of hemorrhagic stroke on CT scans [45,46,47,48], and to predict post-stroke mortality [49,50]. Researchers have also used ML to aid in the acute diagnosis of TIAs and differentiate them from their mimics [51].

(c) Acute Imaging

The use of machine learning, especially deep learning in the field of imaging, has grown exponentially in recent years, leading to improved prediction and diagnosis ability. For cardiovascular disease, ML has been used to aid in the diagnosis and classification of acute and subacute coronary stenosis. Researchers have used ECG data to identify patients with chest discomfort who need urgent revascularization [41]. Other investigators have developed algorithms to make similar diagnoses and classification from myocardial perfusion imaging [26], CT angiography [52], and clinical and laboratory data [53] in emergency settings. 

The two main imaging modalities for the detection of stroke are CT scans and MRI. In the past four years, many studies have been performed in stroke patients that used ML to detect, quantify and subtype ICH on non-contrast CT [46,47,48,54] and MRI [55] in the acute phase. Researchers have also used support vector machine (SVM) algorithms to predict the expansion of hematoma in patients with spontaneous ICH [56]. In hemorrhagic stroke, ML has shown to be promising in detecting large vessel occlusion on CTA [44] and also predicting and quantifying the ischemic core [43,57]. In a different study, Fhager and colleagues implemented binary classification on a broadband microwave imaging technique that can potentially detect ICH outside of dedicated stroke centers [45].

Although advances in the application of machine learning for acute imaging had significant progress in both fields, ML has been used more extensively in the quantification of brain biomarkers when compared to markers from cardiovascular imaging. Nonetheless, the field is at the stage of transitioning to prospective trials and effective implementation at the bedside in multiple settings.

(d) Triaging and Acute Treatment

While diagnosis in cardio-and cerebrovascular fields is one of the first steps after hospital admission, risk stratification during triage can help optimize the available resources and tailor the care management. However, the need for rapid response also requires the tools to interact in real-time with the output from the imaging device and the EHR data. Therefore, the implementation of such tools can be complex and often require coordination at different levels. For instance, the risk of in-hospital cardiac arrest has been predicted using a decision tree [58], while other ML algorithms have been used for risk stratification of chest pain patients using coronary CTA data [52]. These tools, once externally validated and implemented to act in real-time in clinical settings, could help reduce the time for treatment and help save lives.

Using technologies to improve triaging during the acute phase has been more productive in recent years in the cerebrovascular field. ML has been used for recognition and differentiation of ischemic stroke using clinical data [42] and to predict the 90-day mRS score to aid with thrombectomy [59]. MRI data has been used for the classification of ischemic stroke onset time [60] and segmentation and phenotyping of acute ischemic lesions [55]. Researchers have also used ML to estimate ICH volume on CT scan images [47]. The use of ML in triaging stroke patients has escalated further, and authors have discussed the scope and limitations of an ML-based decision support system framework to aid physicians in urgent settings. 

In a real-world environment, initial patient notes can complement pre-event information, if available, for the identification of patients at risk of stroke, and alert the physician to take the guideline-compliant steps to improve the outcome [61]. However, the processing of clinical notes requires advanced natural language processing (NLP) that is carefully tailored for clinical applications. NLP has been mostly applied to reports (such as radiology reports) with promising results [62]; NLP applied to clinical notes can have clinical utility at improving the identification of patients for major vascular events [61]. 

### 3.3. Application of AI in Post-Diagnosis Outcome Prediction and Secondary Prevention

(a) Personalized Treatment

Patient subtyping is a central part of personalized patient care and can be a stand-alone tool to classify patients with similar profiles based on the available information on the patients and their family members. 

Finding clusters of stroke patients can be helpful from the medical perspective as it may lead to the discovery of new patterns and more effective ways to manage a specific condition and its complications. Garg et al. [63] developed an automated stroke subtype classification using radiology and progress reports and showed agreement with the manual TOAST (Trial of ORG 10172 in acute stroke treatment) [64] classification. The challenge of the study remains in its validation in an external cohort. Some other studies are attempting to create a CDSS to help physicians classify stroke subtypes based on limited clinical data. Keerthana [65] used Fuzzy C-Means clustering techniques for the segmentation of brain stroke using MRI images. The study lacked technical details, including the number of cases used in model development and testing. Subtyping in the field of cardiovascular medicine is relatively new, with clinical applications that remain relatively sparse [28,66,67,68,69,70]. Shah et al. predicted the survival of patients with HFpEF using an unsupervised learning model and demonstrated the benefits of deep phenotyping in these patients [71]. The researchers created an unsupervised learning model across 46 different variables to identify intrinsic structures within patients with HFpEF; they identified three distinct groups. The study needs to be replicated in external HFpEF cohorts to demonstrate generalizability. Zhao et al. applied a constrained non-negative tensor factorization approach to classifying patients with the cardiovascular disease based on their longitudinal EHR data [72]. The latter study is unique as it encompasses data from patients ten years before their development of heart disease with the observation of emerging phenotypes of 12,380 cardiovascular diseases. In another study, Ahmad et al. [73] analyzed data from 1619 participants in the HF-ACTION (Heart Failure: A Controlled Trial Investigating Outcomes of Exercise Training) to identify the subtypes of chronic heart failure. The study design excluded patients with incomplete data, thus limiting the true value of the predicting models for clinical applications. Nonetheless, four subtypes were identified, and each patient in the corresponding subtype responded distinctively to exercise therapy. In another study, Schulam et al. [74] used Limestone, a non-negative tensor factorization algorithm, to identify multiple candidate phenotypes of heart failure. Their clinical evaluation results showed the potential ability of Limestone to produce the phenotypes that can identify disease subtypes with potential clinical utility. Panahiazar et al. [75] used clustering techniques to investigate the heart failure patients’ response to therapy. The authors used K-means and hierarchical clustering to group heart failure patients that responded to medication. The similarity assessment of a new patient with each identified cluster could lead to the determination of an appropriate medication plan. The major limitation in these studies remains selection bias, given that in many cases, patients with a poor data footprint are excluded from modeling. However, overall, these examples demonstrated the potential of ML-enabled methods based on patient similarity as assistive tools.

(b) Outcome Prediction

Prediction of outcome after diagnosis was the most extensively investigated application of ML among the categories included in this literature review. Here, the outcomes of interest included, but were not limited to, disease severity, survival, mortality, length of hospitalization, rehospitalization, and recurrence. In patients with confirmed coronary artery disease (CAD), clinical and laboratory data have been used in addition to CTA [17,76], and angiogram [77] to predict cardiovascular events or death with promising results. In one study by Johnson and colleagues, ML algorithms proved superior to CAD reporting and data system (CAD-RADS) scoring in predicting future cardiovascular events and mortality in patients with positive CTA results [17]. In another study, the random forest-based model was shown to better identify patients at risk of 30-day congestive heart failure rehospitalization and 180-day cardiovascular mortality following a percutaneous coronary intervention, compared to conventional methods [78]. Other studies have explored the application of ML in patients admitted for acute coronary syndrome to predict in-hospital mortality [79], 30-day mortality [80], and long-term survival [81,82,83,84]. Duane et al. have proposed a deep learning model using static and dynamic features in 2930 patients with acute coronary syndrome to predict major adverse events in the future [85]. A major study from Sweden used 39 survival predictor variables in 51,943 patients to develop various ML models that could accurately predict two-year survival after the first MI event [82]. At the same time, Pieszo et al. used laboratory values in MI patients to predict long-term mortality, while Kwon and colleagues combined laboratory data with patient demographics to make similar predictions [83,84].

Heart failure is yet another area where ML has shown promising results in the prediction of outcomes [86,87]. In the study published by Kwon et al., machine learning algorithms were able to predict in-hospital and long-term mortality following acute heart failure more effectively than conventional scoring systems [88]. Survival in patients with pulmonary hypertension has also been predicted using ML [89]. Distinguishing between short-term vs. long-term mortality is equally beneficial for the patients and healthcare system as it can help with resource optimization as well as more personalized care [50].

Ischemic and hemorrhagic stroke has been the main focus of cerebrovascular studies with regard to secondary prevention and functional outcome as well as mortality prediction. Researchers used deep learning on acute ischemic stroke imaging features to predict lesion volume [90]. Two different teams of scientists have used ML algorithms to predict three-month functional outcomes following ischemic stroke [91,92]. ML has also been utilized to predict 90-day readmission [93] and one-year recurrence in patients with ischemic stroke [94]. In patients undergoing endovascular treatment for ischemic stroke, ML algorithms did not improve outcome prediction when compared to logistic regression [95]. In hemorrhagic intracranial events, ML has been successful in predicting hematoma expansion [56] and delayed ischemia [96]. 

As such, there has been an increasing number of successful applications of AI in predicting outcomes in cardiovascular and cerebrovascular diseases, raising the question of when these improvements can be evaluated for clinical utility and generalizability to reach patients’ bedsides. In this context, the functional outcome in stroke patients is primarily measured by the modified Rankin Scale (mRS) score [97], while the New York Heart Association (NYHA) classification is used to categorize heart failure patients [98]. Using these scores as features in the machine learning models can be important for training the models. However, the main limiting factor remains the lack of proper reporting of functional classes and the level of missingness in these measurements across the different healthcare systems. Incorporating functional outcomes in a structured form in EHR data to enable easier integration of these measures in machine learning models is an important first step. Better, more consistent, and standardized reporting of functional class scores will ultimately lead to better model predictions.

### 3.4. Application of AI in Rehabilitation

(a) Personalized Treatment

Studies on the use of ML in assisting with rehabilitation have been limited. In heart failure patients, ML helped investigators to classify heart failure patients based on clinical presentation and improve treatment response by directing personalized therapies [99]. In the only cerebrovascular study, researchers used ML to predict activities of daily living in post-stroke patients to better optimize clinical care [100]. Personalized treatment for tertiary prevention is an area with great potential for the application of AI. Rehabilitation in both cardio and cerebrovascular patients has a major financial burden on healthcare systems [101,102]. Innovative use of ML in this field can lead to improved resource optimization and personalized patient experience [103].

(b) Outcome Prediction

Outcome prediction using ML during rehabilitation in cardiovascular studies has been mainly focused on cardiac resynchronization therapy outcomes in patients with heart failure. Researchers have used ML to predict patient response to cardiac resynchronization [104], outcome [105], and mortality [106]. ML has also been used to distinguish different heart failure phenotypes [86] and predict survival with the aid of echocardiography data [16]. In the only cerebrovascular study that we were able to identify, researchers used ML to predict activities of daily living in post-stroke patients to better optimize clinical care [100]. This field has great potential for future studies and trials to improve the recovery and quality of life of patients.

**Table 1:** Cardiovascular studies using artificial intelligence.

Ref., Year—Category **	Study Details	Sample Size	Algorithms
AI and Risk Stratification Modeling			
[10], 2017—1a	Location: United Kingdom Aim: Predicting the first CVD event over 10-years and comparing that with the American College of Cardiology guidelines. Variables: Routine clinical data from family practices Strengths: Prospective; large sample size Limitations: Unbalanced dataset Findings: Highest achieving algorithm was NN: AUC 0.76, predicted 4998/7404 cases (sensitivity 67.5%, PPV 18.4%) and 53,458/75,585 non-cases (specificity 70.7%, NPV 95.7%), correctly predicting 7.6% more patients than the established algorithm	378,256	RF, LR, GBM, NN
[12], 2017—1a	Location: United States Aim: Predict six cardiovascular outcomes in comparison to standard risk scores. Variables: 735 variables from imaging and non-invasive tests, questionnaires, and biomarker panels Strengths: Prospective; included participants from the MESA (Multi-Ethnic Study of Atherosclerosis) [22]; 12-year follow-up; four ethnicities Limitations: Potential cause for biases due to imputation procedure Findings: Age was the most important predictor for all-cause mortality. Fasting glucose levels and carotid ultrasonography measures were important predictors of stroke. CAC was the most important predictor of coronary heart disease and all atherosclerotic cardiovascular disease combined outcomes. Left ventricular structure and function and cardiac troponin-T were among the top predictors for incident heart failure. Creatinine, age, and ankle-brachial index were among the top predictors of AF. TNF-α and IL-2 soluble receptors and NT-proBNP levels were important across all outcomes. Notable facts: ML in conjunction with deep phenotyping improves prediction accuracy in cardiovascular event prediction in an initially asymptomatic population.	6814	RF
[11], 2019—1a, 1b	Location: United States Aim: Predicting of long-term risk of MI and cardiac death in asymptomatic subjects by integrating clinical parameters with CAC, and automated EAT quantification. Variables: Clinical co-variates, lipid panel, risk factors, CAC, aortic calcium, and automated EAT measures Strengths: Prospective; subjects from EISNER trial [23]; 14.5 years follow-up Limitations: Unbalanced data Findings: AUC 0.82; Subjects with a higher ML score had high hazard of suffering events (HR: 10.38, *p* < 0.001); the relationships persisted in multivariable analysis including ASCVD-risk and CAC measures (HR: 2.94, *p* = 0.005). Age, ASCVD-risk, and CAC were prognostically important for both genders. Notable facts: ML used to integrate clinical and quantitative imaging-based variables significantly improves prediction of MI and cardiac death.	1912	XGBoost
[14], 2017—>1a	Location: China Aim: Identifying the association between the clinical reference range of serum HbA1c and TSH, and the risk of CAD in non-diabetic and euthyroid patients. Variables: HbA1c and TSH levels Strengths: Prospective; 10-year follow-up Limitations: Small sample size Findings: Baseline HbA1c and TSH within the reference range were positively associated with CAD risk. No correlation and interaction between the baseline HbA1c and TSH for the development of CAD. The combination of these baselines showed sensitivity of 87.2%, specificity of 92.7%, and accuracy of 92.3% for identifying the participants who will later develop CAD.	538	SVM
[107], 2018—1a	Location: Lebanon Aim: Comparing ANN-based prediction models to the other risk models being used in practice (the Diamond–Forrester and the Morise models). Variables: Imaging-based stress test measures Strengths: Prospective Limitations: Small sample size Findings: Compared to other models, the ANN model had higher discriminatory power (DP) (1.61) for predicting ischemia, 98% negative predictive value, 91% sensitivity, 65% specificity, 26% positive predictive value, and a potential 59% reduction of non-invasive imaging.	486	ANN
[28], 2018—1b, 3a	Location: United Kingdom, Italy, Norway Aim: Discriminating between healthy and HFpEF subjects with impaired functional reserve and identifying new descriptors to better characterize HFpEF syndrome using basal myocardial long-axis velocity patterns at rest and exercise. Variables: Left ventricular long-axis myocardial velocity patterns Strengths: Prospective, 6–60 months survival analysis Limitations: Confounding effects (age, gender) not studied, small sample size Findings: ML-diagnostic zones differed for age, body mass index, six-minute walk distance, B-type natriuretic peptide, and left ventricular mass index. Correlation with diagnosis was 72.6%; ML identified 6% of healthy controls as HFpEF. Blinded reinterpretation of imaging from subjects with discordant clinical and ML diagnoses revealed abnormalities not included in diagnostic criteria.	156	Clustering
[71], 2015—1b, 3a	Location: United States Aim: Identify phenotypically distinct HFpEF categories. Variables: Clinical, laboratory, ECG, and echocardiographic phenotyping (phenomapping) Strengths: Prospective Findings: Phenomapping classified study participants into three risk-stratified groups. Notable facts: A novel classification of HFpEF using phenomapping that can define therapeutically homogeneous patient subclasses.	397	Clustering
[16], 2019—1a, 3a, 4b	Location: United States Aim: Predicting survival after echocardiography. Variables: 90 cardiovascular-relevant ICD-10 codes, age, sex, height, weight, heart rate, blood pressures, LDL, HDL, smoking, physician-reported EF, 57 echocardiographic measurements Strengths: Large sample size Limitations: Retrospective, model derivation from EHR data missing important variables Findings: Overall AUC > 0.82 over common clinical risk scores. RF outperformed LR. RF including all echocardiographic measurements yielded the highest prediction accuracy. Ten variables needed to achieve 96% maximum prediction accuracy, six from echocardiography.	171,510	RF
[17], 2019—3a, 3b	Location: United States Aim: Using ML to develop a model of vessel features to discriminate between patients with and without subsequent death or cardiovascular events and comparing to CAD-RADS. Variables: Four CTA features for each of the sixteen coronary segments Strengths: Comparing four different ML methods Limitations: Low MI incidence leading to possible misclassification bias Findings: ML all-cause mortality AUC = 0.77; ML CAD deaths AUC = 0.85. For starting statin therapy (NNT = 45), use of ML score ensures 93% of patients with events will be administered the drug; compared to 69% with CAD-RADS. Notable facts: Compared to CAD-RADS, ML better discriminated patients who subsequently experienced an adverse event from those who did not.	6892	Best models: bootstrap-aggregated DTE, KNN,
[13], 2018—1a, 1c	Location: United States Aim: Developing a risk calculator for CAD incidence to aid initiation of statin therapy. Variables: Same as ACC/AHA risk calculator Strengths: Model training by 13-year follow-up data from MESA cohort [22] and validation by FLEMENGHO cohort [108] Limitations: Retrospective Findings: ML Risk Calculator recommended only 11.4% to take statin, and only 14.4% of “Hard CVD” events occurred in those not recommended statin, resulting in sensitivity 0.86, specificity 0.95, and AUC 0.92. Notable facts: ML Risk Calculator outperformed the ACC/AHA Risk Calculator by recommending less drug therapy yet missing fewer events.	10,291	SVM
[109], 2019—1a, 1b	Location: Iran Aim: Compare ANN and SVM algorithms for predicting CAD. Variables: 25 variables affecting CAD including laboratory values Strengths: Data collected from three hospitals Limitations: Retrospective; no detail provided regarding missingness, or lack thereof Findings: SVM model had higher AUC, higher sensitivity, higher Hosmer–Lemeshow test’s result and lower MAPE compared to ANN. Variables affecting CAD yielded better goodness of fit in SVM model and provided more accurate result than ANN.	1324	ANN, SVM
AI-enabled Diagnostic Studies			
[76], 2016—3b	Location: Multi-national Aim: Predicting five-year all-cause mortality in patients undergoing CCTA and comparing to existing prediction algorithms. Variables: 25 clinical and 44 CCTA parameters, SSS, SIS, DI, number of segments with non-calcified, mixed or calcified plaques, age, sex, gender, standard cardiovascular risk factors, and FRS Strengths: Data from CONFIRM registry [110]; large sample size Limitations: Selection bias; only LogitBoost was evaluated for efficacy. Findings: ML exhibited a higher area-under-curve compared with the FRS or CCTA severity scores alone (SSS, SIS, DI) for predicting all-cause mortality (ML: 0.79 vs. FRS: 0.61, SSS: 0.64, SIS: 0.64, DI: 0.62; *p* < 0.001). Notable facts: ML combining clinical and CCTA data was found to predict five-year all-cause mortality significantly better than existing clinical or CCTA metrics alone.	10,030	LogitBoost
[77], 2019—3a, 3b	Location: Korea Aim: Developing an angiography-based supervised ML algorithm with five-fold cross-validation to classify coronary lesions based on fractional flow reserve (≤0.80 vs. >0.80). Variables: 24 computed angiographic features based on the diameter plot and four clinical features (age, sex, body surface area, and involve segment) Strengths: Randomized controlled trial; external validation in 79 patients Limitations: Data, analytic methods, and study materials not available to other researchers; model limited to left main disease, side branch, and diffuse and tandem lesions Findings: ML model predicted fractional flow reserve ≤ 0.80 with overall diagnostic accuracy of 78% (AUC = 0.84). Using 12 main angiography features, the ML predicted fractional flow reserve ≤ 0.80 in the test set with sensitivity of 84%, specificity of 80%, and overall accuracy of 82% (AUC = 0.87). The averaged diagnostic accuracy in bootstrap replicates was 81% (AUC = 0.87). External validation showed accuracy of 85% (AUC = 0.87).	1501	XGBoost
[39], 2017—2a, 2b	Location: Canada Aim: Automating the diagnosis of STEMI at the time of first contact with healthcare system and pre-hospital CCL activation. Variables: ECG reading data Limitations: Retrospective analysis of real-time automated diagnosis; only ECG data used; small sample size Findings: Algorithm modification resulted in a 42% relative decrease in the rate of inappropriate activations (12% vs. 7%) without a significant effect on treatment delay.	466	Automated STEMI diagnosis and “physician-less” CCL activation
[41], 2019—2b, 2c, 2e	Location: Japan Aim: Making an AI prediction model for the need for urgent revascularization from 12-lead ECG in patients presenting with chest pain in the ER. Variables: ECG reading data Limitations: Retrospective; only ECG data used, small sample size Findings: Predictive value of the c-statistics 0.88 (95% CI 0.84–0.93) for detecting patients who required urgent revascularization.	362	LSTM
AI in Outcome Prediction/Prognosis			
[89], 2017—3b	Location: United Kingdom Aim: Predicting patient survival in pulmonary hypertension using 3D patterns of systolic cardiac motion. Variables: Conventional imaging; hemodynamic, functional, and clinical markers; 3D motion pattern of right ventricle Strengths: Prospective Limitations: Limited patient selection including non-congenital cases of PH. Model trained to measure excursion rather than contractility. Findings: Survival prediction AUC 0.73; difference in median survival time between high- and low-risk groups was 13.8 years.	256	Supervised ML using nested multivariable risk prediction
[111], 2019—3a	Location: United States Aim: Testing generalizability and precision in imaging biomarker analysis by comparing scan:rescan data. Variables: MR-measured left ventricular chamber volumes, mass, and ejection fraction Strengths: Prospective Limitations: Data from five institutions, but scans performed at the same institution; one-week interval between scans limited the ability to assess long-term changes Findings: Expert, trained junior, and automated scan:rescan precision were similar (coefficient of variation 6.1 vs. 8.8). Automated analysis was 186× faster than humans.	110	CNN
[82], 2017—3b	Location: Sweden Aim: Predicting two-year survival vs. non-survival after first MI. Variables: 39 survival predictors Strengths: Large sample size Limitations: Retrospective Findings: SVM had the highest performance (AUC = 0.845, PPV = 0.280, NPV = 0.966) outperforming Boosted C5.0 (AUC = 0.841), but not significantly higher than LR or RF. Models converged to the point of algorithm indifference with increased sample size and predictors.	51,943	SVM, RF, LR, Boosted C5.0
[86], 2018—3b, 4b	Location: Sweden Aim: Using mixture of supervised and unsupervised approach to predict outcome and identify distinct phenotypes of heart failure. Variables: Demographic, clinical, laboratory, and medication data Strengths: Large sample size Limitations: Retrospective Findings: RF demonstrated excellent calibration and discrimination for survival (C-statistic = 0.83) whereas LVEF did not (C-statistic = 0.52). Cluster analysis using the eight highest predictive variables identified four clinically relevant subgroups of HF with marked differences in one-year survival.	44,886	RF, K-means clustering
[79], 2017—3b	Location: United States Aim: Modeling all-cause in-hospital mortality in women admitted with STEMI. Variables: 11 variables for LR; 32 variables for full RF model; 17 variables for reduced RF model Strengths: Model validation using external cohort of 13,361 patients Limitations: Retrospective; class imbalance (in-hospital mortality in 11% of patients) Findings: Internal validation C-index was 0.84, 0.81, and 0.80 for the LR, full, and reduced RF models, respectively. External validation C-index was 0.84, 0.85, and 0.81 for year 2011, and 0.82, 0.81, and 0.81 for the year 2013 for the LR, full, and reduced RF models, respectively. Notable facts: RF was comparable to LR in predicting in-hospital mortality in women with STEMI.	12,047	LR and RF
[84], 2019—3b	Location: Korea Aim: DL-based risk stratifying mortality of patients with acute MI. Variables: Initial demographic and laboratory data Strengths: Large sample size; data from the Korean working group of myocardial infarction registry (network of 59 hospitals) Limitations: Retrospective Findings: AUC for STEMI = 0.905. AUC for NSTEMI = 0.870. DL predicted 30.9% of patients more accurately than conventional scores. During the six-month follow-up, the DL-defined high-risk group had a significantly higher mortality rate than the low-risk group (17.1% vs. 0.5%).	22,875	DL, LR, RF
[58], 2019—2d, 3b	Location: China Aim: Identify in-hospital cardiac arrest in hospitalized patients with acute coronary syndrome. Variables: Seven explanatory variables: VitalPAC Early Warning Score (ViEWS), fatal arrhythmia, Killip class, cardiac troponin I, blood urea nitrogen, age, and diabetes Limitations: Possibility of selection bias Findings: Sensitivity = 0.762; Specificity = 0.882; AUC = 0.844; a 10-fold cross-validated risk estimate = 0.198; optimism-corrected AUC = 0.823. Notable facts: The developed DT model may provide healthcare workers with a practical bedside tool and could positively impact decision-making in deteriorating patients with ACS.	656	DT
[78], 2019—3b	Location: United States Aim: Identify patients at risk of death or CHF rehospitalization after PCI. Variables: 52 features at admission to predict in-hospital mortality; 358 features at discharge to predict CHF readmission Strengths: Large sample size Limitations: Retrospective; high missingness level in certain features causing high data sparsity Findings: RF prediction of in-hospital mortality AUC = 0.925. RF outperformed LR for predicting 30-day CHF readmission (AUC: 0.90 vs. 0.85) and 180-day cardiovascular death (AUC: 0.88 vs. 0.81).	11,709	RF
[88], 2019—3b	Location: Korea Aim: Developing and validating a deep-learning-based AI algorithm for predicting mortality of acute HF. Variables: Demographics, treatment and medication, laboratory, ECG and echocardiography findings, final diagnosis, clinical outcome during hospital stay, and 12-month prognosis Strengths: Multi-center study; large sample size Limitations: Retrospective Findings: AUC of the DL was 0.880 for predicting in-hospital mortality, which outperformed other machine learning models. For predicting 12- and 36-month endpoints, DL had an AUC of 0.782 and 0.813, respectively. During the 36-month follow-up, the high-risk group, defined by the DL, had a significantly higher mortality rate than the low-risk group.	6924	DNN, RF, LR, SVM, BN
[53], 2019—2c, 2d	Location: Korea Aim: Using ML to predict ACS requiring revascularization in patients presenting with early-stage angina-like symptoms. Variables: 20 features relevant to ACS Strengths: Large sample size Limitations: Retrospective; inaccuracy in checking the vulnerable plaque burden of all coronary arteries Findings: AUC = 0.860 for the prediction of ACS requiring revascularization. A reliable prediction of 2.60% of non-ACS patients was made with a specificity of 1.0 to only receive medical therapy.	5882	SVM, LDA
[87], 2019—3b	Location: United States Aim: Using a ML algorithm to predict mortality in HF patients. Variables: Eight variables: diastolic blood pressure, creatinine, blood urea nitrogen, hemoglobin, white blood cell count, platelets, albumin, and red blood cell distribution width Strengths: Large sample size Limitations: Retrospective; selection bias due to excluding significant number of patients with missingness Findings: The risk score developed by DT accurately discriminated between low and high-risk of death with an AUC of 0.88. External validation in two separate HF populations gave AUCs of 0.84 and 0.81.	5822	DT
[83], 2019—3b	Location: United Kingdom Aim: Predicting long-term mortality after ACS using laboratory values. Variables: Hematological indices and inflammation markers Strengths: Large sample size Limitations: Imputation for the ML was performed using mean of all observations, the latter is typically not ideal since missing in EHR data tend to be not-at-random Findings: The model achieved a c-statistic of 0.89 for in-hospital mortality. C-statistic was 0.77 for six-month mortality. Red cell distribution width (HR 1.23) and neutrophil to lymphocyte ratio (HR 1.08) showed independent association with all-cause mortality in multivariable Cox regression.	5053	XGBoost
[85], 2019—3b	Location: China Aim: Developing a DL model to predict major adverse cardiac events after ACS. Variables: 232 static feature types and 2194 dynamic feature types. Strengths: Large sample size; comparison to previous models Limitations: Retrospective; missing values (up to 30%) were imputed using median of all the observations; variables with more than 30% missing were excluded Findings: The best model presented had an AUC of 0.713 and an accuracy of 0.764. Notable facts: The proposed model adapted to leverage dynamic treatment information in EHR data boosted the performance of major adverse cardiac event prediction for ACS.	2930	RNN
[80], 2017—3b	Location: Israel Aim: Predicting mortality at 30-days in STEMI patients and to compare these to the conventional validated risk scores. Variables: 54 variables; performance of most models plateaued with 15 variables Strengths: Large sample size Limitations: Retrospective Findings: ML models AUC range: 0.64 to 0.91. The best models had similar or better performance compared to standard scoring methods. Top predictors were creatinine, Killip class on admission, blood pressure, glucose level, and age. Notable facts: The algorithms selected showed competence in prediction across an increasing number of variables.	2782	NB, DT, LR, rules-based classification tree, RF, Adaptive Boosting
[112], 2018—3a	Location: Canada Aim: Assessing the prognostication of NN in HF patients using CPET data as opposed to using summary indicators alone. Variables: Detailed CPET data Strengths: Using various ML models Limitations: Retrospective Findings: NN incorporating breath-by-breath data achieved the best performance (AUC = 0.842). All models outperformed summary indices (AUC ≤ 0.800). When compared with the CPET risk score (AUC = 0.759), the top-performing model obtained a net reclassification index of 4.9%. Notable facts: The current practice of considering summary indices in isolation fails to realize the full value of CPET data. Higher data resolution leads to improved prediction.	1434	LASSO, NN
[81], 2020—3b	Location: China Aim: Using ML to predict one-year mortality rate of anterior STEMI patients and comparing to conventional risk scores. Variables: 59 features; including all features as opposed to top 20 provided better performance Strengths: Using six different ML algorithms Limitations: Retrospective Findings: AUC of ML models ranged from 0.709 to 0.942. XGBoost achieved the highest accuracy (92%), specificity (99%) and f1 score (0.72) for predictions with the full variable model. After feature selection, XGBoost still obtained the highest accuracy (93%), specificity (99%) and f1 score (0.73).	1244	NB, LR, KNN, DT, RF and XGBoost
[105], 2019—4b	Location: United States Aim: Using ML on EHR data to predict CRT outcome. Variables: Demographics, laboratory values, medications, clinical characteristics, and past health services utilization, bigrams (i.e., two-word sequences) in EHR data Strengths: Comparing various ML models Limitations: No distinction between the type of CRT implant. Findings: The final model identified 26% of patients having a reduced benefit from the CRT device at a PPV of 79% (model performance: Fβ (β = 0.1): 77%; recall 0.26; precision 0.79; accuracy 0.65). Notable facts: A ML model that leveraged readily available EHR data and clinical notes identified a subset of CRT patients who may not benefit from CRT before the procedure.	990	LR, SVM, RF and GBM
[113], 2019—1a	Location: Japan Aim: Assessing stroke risk by ML using integrated risk factors. Variables: 47 features comprised of 13 conventional risk factors and 34 carotid ultrasound image-based phenotypes (carotid intima-media thickness, carotid plaque and carotid artery stenosis) Strengths: Using integrated risk factors Limitations: Retrospective; small sample size; data imbalance (12 high-risk patients vs. 190 low-risk patients) Findings: ML with integrated risk factors (AUC = 0.80) showed an improvement of ~18% against conventional ML (AUC = 0.68). Notable facts: ML model integrated with the event-equivalent gold standard as percentage stenosis is powerful and offers low cost and high-performance stroke risk assessment.	202	RF
AI in Treatment Strategies			
[99], 2018—3a, 4a	Location: Multi-national Aim: Using ML to phenotypically classify a heterogeneous HF cohort and aid in optimizing the rate of responders to specific therapies. Variables: 50 variables including clinical parameters, biomarker values, and measures of left and right ventricular structure and function Strengths: Data from MADIT-CRT trial [114]; randomized cohort Limitations: Possibility of selection bias; results confined to a selected population of HF patients enrolled in a clinical trial with robust inclusion/exclusion criteria Findings: Four phenogroups identified, significantly different in the primary outcome occurrence. Two phenogroups included a higher proportion of known clinical characteristics predictive of CRT response and were associated with a substantially better treatment effect of CRT-D on the primary outcome (HR = 0.35 and HR = 0.36) than observed in the other groups. Notable facts: By integrating clinical parameters and full heart cycle imaging data, unsupervised ML can provide a clinically meaningful classification of a phenotypically heterogeneous HF cohort and might aid in optimizing the rate of responders to specific therapies.	1106	Multiple Kernel Learning, K-means clustering
[104], 2019—4b	Location: United States Aim: Develop and compare ML models to predict response to CRT. Variables: Nine variables; QRS morphology, QRS duration, New York Heart Association classification, left ventricular ejection fraction and end-diastolic diameter, sex, ischemic cardiomyopathy, AF, and epicardial left ventricular lead Strengths: Multi-center study comparing various ML models Limitations: Retrospective Findings: The best ML model was a naïve Bayes classifier. On the testing cohort, ML demonstrated better response prediction than guidelines (AUC 0.70 vs. 0.65) and greater discrimination of event-free survival (concordance index, 0.61 vs. 0.56). The fourth quartile of the ML model had the greatest risk of reaching the composite end point, whereas the first quartile had the least (hazard ratio, 0.34).	925	Supervised ML
[106], 2018—4b	Location: United States Aim: Using ML to predict all-cause mortality or heart failure hospitalization 12 months post-CRT. Variables: 45 features: demographics, physical characteristics, heart failure, LV assessment, ECG, medical history, medication class Strengths: Used data from COMPANION trial [115] Limitations: Possibility of selection bias; only class III and IV HF patients enrolled with specific inclusion/exclusion criteria Findings: RF model produced quartiles of patients with an eight-fold difference in survival between those with the highest and lowest predicted probability for events (hazard ratio, 7.96). The model discriminated the risk of the composite end point of all-cause mortality or heart failure hospitalization better than conventional methods.	1076	Multiple models with RF producing best results
AI-enabled Diagnostic Imaging Studies			
[24], 2018—1b	Location: United States Aim: Determining the diagnostic performance of cPSTA in assessing CAD in patients presenting with chest pain who had been referred by their physician for coronary angiography. Variables: cPSTA recorded signals Strengths: Prospective Limitations: Small sample size Findings: The machine-learned algorithm had a sensitivity of 92% and specificity of 62% on blind testing in the verification cohort. The NPV was 96%. Notable facts: Resting cPSTA may have comparable diagnostic utility to functional tests currently used to assess CAD without requiring cardiac stress (exercise or pharmacological) or exposure of the patient to radioactivity.	606	Elastic net
[25], 2018—1b, 3a	Location: Multi-national Aim: Predicting lesion-specific ischemia by invasive FFR using an integrated ML ischemia risk score from quantitative plaque measures from CCTA. Variables: Quantitative CTA data: stenosis, NCP, low-density NCP (LD-NCP), calcified and total plaque volumes, contrast density difference (maximum difference in luminal attenuation per unit area) and plaque length Strengths: Multi-center data from NXT trial [116] Limitations: Small sample size; plaque findings were not confirmed by invasive intravascular ultrasound Findings: Information gain for predicting ischemia was highest for contrast density difference (0.172), followed by LD-NCP (0.125), NCP (0.097), and total plaque volumes (0.092). ML had higher AIUC (0.84) than individual CTA measures, including stenosis (0.76), LD-NCP volume (0.77), total plaque volume (0.74) and pre-test likelihood of CAD (0.63).	254	LogitBoost
[15], 2020—1a	Location: Multi-national Aim: Evaluate the prognostic value of fully automated DL-based EAT volume and attenuation quantified from non-contrast cardiac CT. Variables: Non-contrast cardiac CT scan data, inflammatory biomarkers Strengths: Data from the EISNER trial [23] Limitations: Long-term follow-up not obtained Findings: Increased EAT volume and decreased EAT attenuation were independently associated with MACE. CAD risk score, CAC, and EAT volume were associated with increased risk of MACE (hazard ratio: 1.03, 1.25, and 1.35). EAT attenuation was inversely associated with MACE (hazard ratio: 0.83, Harrell C statistic: 0.76). MACE risk progressively increased with EAT volume ≥ 113 cm^3^ and CAC ≥ 100 AU; highest in subjects with both. EAT volume correlated with inflammatory biomarkers; EAT attenuation inversely related to inflammatory biomarkers.	2068	DL
[117], 2018—1a	Location: Multi-national Aim: Investigating whether a ML score, using only plaque stenosis and composition information from the 16 coronary segments, has better predictive accuracy compared to the traditional CCTA based risk scores. Variables: 16 segment based coronary stenosis (0%, 1–24%, 25–49%, 50–69%, 70–99% and 100%) and composition (calcified, mixed and non-calcified plaque) derived from CCTA Strengths: Data from CONFIRM registry [110] Findings: ML-based approach showed better AUC for event discrimination (0.771) vs. other scores (ranging from 0.685 to 0.701). Improved risk stratification was the result of down-classification of risk among patients that did not experience events (non-events).	8844	XGBoost
[26], 2018—1b, 1c, 2c	Location: Multi-national Aim: Evaluating DL-based automatic prediction of obstructive disease from MPI, compared with TPD. Variables: MPI recorded data Strengths: Multi-center study Limitations: Retrospective; degree of stenosis from invasive angiography was interpreted visually Findings: AUC for DL was higher than for TPD (per patient: 0.80 vs. 0.78; per-vessel: 0.76 vs. 0.73). Sensitivity per patient improved from 79.8% (TPD) to 82.3% (DL), and per-vessel sensitivity improved from 64.4% (TPD) to 69.8% (DL).	1018	DCNN
[52], 2018—2c, 2d	Location: United States Aim: Evaluating the effectiveness of using Computer-Aided Diagnosis in the triage of low to intermediate risk emergency chest pain patients with CCTA. Variables: Data from 64 and 320 slice CCTA scanners Strengths: Looking at 30-day outcome Limitations: Retrospective Findings: Sensitivity: 85%; specificity: 50.6% and 56.5% for the 64 and 320 slice scanners. NPV: 97.8 and 97.1 for the 64 and 320 slice scanners. AUC: 0.6794 and 0.7097 for the 64 and 320 slice scanners. Software unable to read 18% of the cases.	923	Computer aided diagnosis software
[118], 2018—2c	Location: Multi-national Aim: Improving diagnostic performance of CTA to potentially reducing the number of unnecessary referrals for invasive coronary angiography. Variables: 28 variables from CTA data Strengths: Multi-center Limitations: Retrospective; possibility of selection bias due to the inclusion of patients with the disease only Findings: ML-FFR (AUC = 0.84) and CFD-FFR (AUC = 0.84) outperformed visual CTA (AUC = 0.69). Per-vessel and per-patient diagnostic accuracy improved 78% and 85%, respectively. ML-FFR correctly reclassified 73% of false-positive CTA results. Notable facts: On-site ML-FFR improves the performance of CTA by correctly reclassifying hemodynamically nonsignificant stenosis and performs equally well as CFD-FFR.	351	NN
[27], 2017—1b, 1c	Location: United States Aim: Evaluating the incremental benefit of ML-powered resting myocardial CTP over coronary CT stenosis for predicting ischemia Variables: CCTA and FFR data Strengths: Data from DeFACTO study [119] Limitations: Small sample size Findings: Accuracy, sensitivity, specificity, PPV, and NPV of resting CTP were 68.3%, 52.7%, 84.6%, 78.2%, and 63.0%, respectively, for predicting ischemia. Addition of resting CTP improved discrimination (AUC = 0.75) and reclassification (net reclassification improvement: 0.52) of ischemia compared to CT stenosis alone (AUC = 0.68). Notable facts: The addition of resting CTP analysis acquired from ML techniques may improve the predictive utility of significant ischemia over coronary stenosis.	252	Gradient boosting classifier

** Category definition: *Category 1*: Application of AI in pre-diagnosis modeling: primary prevention (1a: Risk Estimation, 1b: Clustering/patient profiling before the event, 1c: Care gap identification and personalized prevention, 1d: Personalized prevention). *Category 2*: Application of AI in diagnosis and acute-phase treatment (2a: EMS proper referral, 2b: Acute Diagnosis, 2c: Acute Imaging, 2d: Triaging and Acute Treatment). *Category 3*: Application of AI in post-diagnosis outcome prediction and secondary prevention (3a: Personalize Treatment, 3b: Outcome prediction/effect disposition). *Category 4*: Application of AI in rehabilitation (4a: Personalize Treatment, 4b: Outcome Prediction). Abbreviations: ACM: all-cause mortality; ACS: acute coronary syndrome; AF: atrial fibrillation; ANN: artificial neural networks; AUC: area under the receiver operating characteristic curve; BN: Bayesian network; CPET: cardiopulmonary exercise testing; CAC: coronary artery calcium score; CAD: coronary artery disease; CAD-RADS: coronary artery disease reporting and data system; CCTA: coronary computed tomographic angiography; CTA: computed tomographic angiography; CCL: cardiac catheterization laboratory; CDS: clinical decision support; CFD: computational fluid dynamics; CHF: congestive heart failure; CHD: coronary heart disease; CNN: convolutional neural network; CONFIRM: Coronary CT Angiography Evaluation For Clinical Outcomes: An International Multi-center; cPSTA: cardiac phase space tomography analysis; CRT: cardiac resynchronization therapy; CTP: computed tomography perfusion; CVD: cardiovascular disease; DL: deep learning; DCNN: deep-learning convolution neural network; DI: modified Duke index; DT: decision tree; DTE: decision tree ensembles; EAT: epicardial adipose tissue; EMS: emergency medical services; ER: emergency room; FFR: fractional flow reserve; FLEMENGHO: Flemish Study of Environment Genes and Health Outcomes; FRS: Framingham risk score; GBM: gradient boosting machines; HCM: hypertrophic cardiomyopathy; HF: heart failure; HFpEF: heart failure with preserved ejection fraction; KNN: k-nearest neighbors; LASSO: least absolute shrinkage and selection operator; LDA: linear discriminant analysis; LR: linear regression; MACE: major adverse cardiac events; MESA: Multi-Ethnic Study of Atherosclerosis; MI: myocardial infarction; ML: machine learning; NB: Naïve Bayesian; NCP: non-calcified plaque; NN: neural networks; PCA: principal components analysis; PCI: percutaneous coronary intervention; PH: pulmonary hypertension; PPV: positive predictive value; RF: random forest; SCD: sudden cardiac death; SIS: segment involvement score; SSS: segment stenosis score; STEMI: ST-elevation MI; SVM: support vector machine; TSH: thyrotropin; TPD: total perfusion deficit.

**Table 2:** Cerebrovascular studies using artificial intelligence.

Ref., Year—Category **	Study Details	Sample Size	Algorithms
AI and Risk Stratification Modeling			
[18], 2019—1a	Location: China Aim: Proposed a new feature selection method to select important risk factors for detecting ischemic stroke. Variables: 24 blood test features and four demographic features Limitations: Single-center study Findings: Top nine features selected. Sensitivity: 82.7%, specificity: 80.4%, classification accuracy: 81.5%, Youden index: 0.63.	792	Weighting and ranking-based hybrid feature selection (WRHFS)
[19], 2017—1a	Location: China Aim: Build 2-year thromboembolism prediction models for AF patients, Variables: Chinese AF Registry data Strengths: Large dataset, two-year follow-up Limitations: Retrospective; design of the preprocessing and imputation strategy could lead to bias results and model overfitting Findings: AUC: 0.71–0.74. Notable facts: Model superior to previous thromboembolism prediction models.	3535	LR, Cox, NB, CART, RF
[20], 2018—1a	Location: China Aim: Build one-year ischemic stroke prediction models for AF patients. Variables: Chinese AF Registry data Strengths: Large dataset Limitations: Retrospective; highly imbalanced dataset (3.8% rate of stroke at one-year) Findings: AUC: 0.714. Notable facts: Boots-wrapper can balance model discrimination and statistical significance of features for developing AF stroke prediction models.	3736	Bootstrap-based wrapper for feature selection
[21], 2019—1a	Location: Taiwan Aim: Develop a predictive model to estimate three-year risk of ischemic stroke in the general population. Variables: Insurance claim data Strengths: Large sample size; model maintained high predictability five years after being developed. Limitations: Retrospective Findings: AUC: 0.920 (95% CI, 0.908–0.932) in testing dataset 1 and 0.925 (95% CI, 0.914–0.937) in testing dataset 2. Sensitivity and specificity were 80.3–92.5% and 79.8–87.5% for testing dataset 1; 83.7–91.8% and 79.9–87.5% for testing dataset 2. Notable facts: DNN algorithm is capable of obtaining a high performing model for assessment of ischemic stroke risk.	840,487	DNN
[93], 2019—3b	Location: China Aim: Identify the ischemic stroke readmission risk factors and establish a 90-day readmission prediction model for first-time ischemic stroke patients. Variables: Clinical data Strengths: Compared predictions at various follow-up periods Limitations: Retrospective; imputation of missing values is not discussed; dataset highly imbalanced (8.6% readmission rate) Findings: Standard AUC: 0.782 (0.729–0.834); best time-dependent AUC : 0.808 in 54 days. Notable facts: XGboost model obtained a better risk prediction for 90-day readmission for first-time ischemic stroke patients than the LR model.	6070	XGBoost, LR
AI-enabled Diagnostic Studies			
[42], 2017—2b, 2d	Location: USA Aim: Recognize acute cerebral ischemia and differentiate that from stroke mimics at the initial examination. Variables: Clinical data Strengths: Prospective; ten-fold cross-validation Limitations: Stroke subtypes not classified Findings: Sensitivity: 80.0% (95% CI, 71.8–86.3); specificity: 86.2% (95% CI, 78.7–91.4); median precision: 92% (95% CI, 88.7–95.3). Notable facts: ANN can be an effective tool to recognize ACI and differentiate it from stroke mimics at the initial examination.	260	ANN
[49], 2019—2b	Location: Korea Aim: Detecting stroke and modeling mortality; stroke definition based on ICD code. Variables: Gender, age, type of insurance, admission type, brain surgery required, region, LOS, hospital location, number of hospital beds, stroke type, and CCI Strengths: Large sample size Limitations: Retrospective Findings: AUC: 83.48%. Notable facts: A scaled PCA/deep neural network approach can be used by both patients and doctors to prescreen for possible stroke.	15,099	PCA, DNN, RF, GNB, KNNC, SVM, ADB
[45], 2019—2b, 2c	Location: Sweden Aim: Detecting intracranial bleeding using simulated microwave transmission data, leveraging numerical simulation based on 3D finite-difference time-domain modeling. Variables: Computational model from an anatomical tissue of a human head; bleeding model is simplified representation of intracranial bleeding (resembling acute phase) Strengths: Simulated cohort Limitations: Retrospective Findings: With a sample size that approached 1000 subjects, classification results characterized as AUC > 0.9. Notable facts: Results indicate very high sensitivity and specificity.	Synthetic cohort	BC
[94], 2019—3b	Location: China Aim: Identifying high-risk TIA or minor stroke patients (recurrent ischemic stroke within one year). Variables: Demographics, clinical and imaging data Strengths: Patients with stroke or TIA mimics were excluded Limitations: Retrospective; downsampling the majority class applied to address data imbalance Findings: ANN median sensitivity: 75%; specificity: 75%; accuracy: 75%; c statistic: 0.77. Notable facts: ANN model outperformed SVM and Naïve Bayes.	451	ANN, SVM, NB
[46], 2019—2b, 2c	Location: USA Aim: Detecting acute intracranial hemorrhage on head CT scans using DL. Variables: CT scan data Strengths: Large sample size Limitations: Retrospective Findings: AUC: 0.991 ± 0.006. Notable facts: Demonstrated end-to-end network that performs joint classification and segmentation with examination-level classification comparable to experts, in addition to robust localization of abnormalities.	4596	FCN
[60], 2018—2d	Location: USA Aim: Classifying acute ischemic stroke onset time. Variables: MRI features Strengths: Extracted hidden representations from the MR perfusion-weighted images Limitations: Retrospective; possibly selection bias due to missingness; only ~10% of patients had sufficient information to be included in the study Findings: AUC: 0.68. Notable facts: Classification significantly improved over current clinical methods, demonstrating the potential benefit of using ML methods in TSS classification.	105	FLIRT, SMR, SVM, RF, GBRT
AI in Outcome Prediction/Prognosis			
[96], 2018—3a, 3b	Location: USA Aim: Developing and validating model for delayed cerebral ischemia after subarachnoid hemorrhage. Variables: Age, sex, Hunt-Hess grade, modified Fisher Scale (mFS), and Glasgow Coma Scale (GCS) Strengths: Prospective Limitations: Possibility of selection bias; patients with missingness excluded Findings: Standard grading scale (mFS): AUC 0.58; combined demographics and grading scales: AUC 0.60; random kernel derived physiologic features: AUC 0.74; combined baseline and physiologic features with redundant feature reduction: AUC 0.77.	488	PLS, linear & kernel SVM
[91], 2019—3b	Location: Korea Aim: Predict the three-month outcomes (mRS) in ischemic stroke patients. Variables: Clinical data Strengths: Large sample size Limitations: Retrospective Findings: DNN AUC was significantly higher than that of the ASTRAL score (0.888 vs. 0.839; *p* < 0.001) when 38 variables were used. When only the six variables from the ASTRAL score were used in the ML models, there was no significant difference in performance.	2604	DNN, RF, LR
[95], 2018—3a, 3b	Location: Netherlands Aim: Predicting the outcome of endovascular treatment for acute ischemic stroke. Variables: 53 baseline variables and 30 treatment variables Strengths: Large sample size Limitations: Retrospective Findings: Range mean AUC = 0.88–0.91 with a negligible difference of mean AUC (0.01; 95%CI: 0.00–0.01) between best performing ML algorithm (RF) and best performing LR model. Notable facts: In large vessel occlusion patients, ML did not outperform LR models in predicting reperfusion and three-month functional independence after endovascular treatment. Radiological outcome was more difficult to predict than clinical outcome at time of admission.	1383	Super Learner (ensemble method), RF, SVM, ANN
[92], 2019—3b	Location: Switzerland Aim: Predicting the outcome (mRS > 2) at 90 days in patients with acute ischemic stroke. Variables: Biomarkers available at admission, NIHSS score Limitations: Retrospective Findings: XGB and GBM AUC = 0.746 and 0.748; improved after adding NIHSS and feature selection to 0.884 and 0.877, respectively. Notable facts: DT-based GBMs can predict the recovery outcome of stroke patients at admission with a high AUC.	512	XGB, GBM
[120], 2018—3a	Location: China Aim: Identifying a neurological deterioration prognostic model, based on dehydration equations. Variables: age, sex, laboratory values, and vascular risk factor data Strengths: Feature selection by the Boruta algorithm Limitations: Retrospective Findings: After decreasing the number of variables from 18 to 5, the specificity of test samples for the SVM prediction model increased from 44.1% to 89.4%, and the AUC increased from 0.700 to 0.927. Notable facts: SVM algorithms can be used to establish a prediction model for dehydration-associated ND, with good classification results.	382	SVM
[100], 2018—4a, 4b	Location: Taiwan Aim: Prediction of Barthel index (BI) status at discharge to optimize care of post-stroke patients. Variables: 15 rehabilitation assessments variables Limitations: Retrospective; patients were excluded (43) due to incomplete data; ratio of men to women was 2:1 Findings: LR and RF algorithms performed higher (AUC = 0.79) than SVM (AUC = 0.77). Mean absolute error of SVM and LR in predicting BI at discharge were 9.86 and 9.95, respectively. Notable facts: The proposed ML-based method provides a promising and practical computer-assisted decision-making tool for predicting ADL in clinical practice.	313	SVM, RF, LR
AI in Treatment Strategies			
[35], 2018—1a, 1b, 1c	Location: Switzerland Aim: Investigating whether a CDS tool for stroke prevention integrated in EHR could improve adherence to guidelines in patients with AF in a PCP setting. Strengths: Randomized clinical trial; the analysis was carried out in a catchment area with high baseline adherence rate Findings: No difference observed in the incidence of stroke, TIA, or systemic thromboembolism in CDS group vs. control group. CDS group had a lower incidence of significant bleeding.	444,347	CDS system
[59], 2019—2d	Location: USA Aim: Develop a regression tree model predict 90-day modified Rankin Scale (mRS) scores to aid with ET. Variables: Elderly patients defined as ≥ 80 years of age Strengths: Retrospective and prospective; the model validated using an independent prospective cohort (36) of patients presenting to the same institution Limitations: Small sample size Findings: Sensitivity: 89.36%; specificity: 89.66%; AUC: 0.952. Notable facts: Algorithm is useful to determine which patients to exclude from ET, and has been implemented in an online calculator for public use.	110	Regression tree
AI-enabled Diagnostic Imaging Studies			
[47], 2018—2b, 2c, 2d	Location: USA Aim: Detecting and quantifying intraparenchymal, epidural, subdural and subarachnoid hemorrhages on non-contrast CT (NCCT) and estimating hemorrhage volume. Variables: Training set: 10,159 NCCT examinations, 901 of which contained hemorrhage. Testing set: 682 prospective NCCT examinations, 82 of which contained hemorrhage Strengths: Retrospective and prospective evaluation Findings: Hemorrhage detection accuracy: 0.970, AUC: 0.981, sensitivity: 0.951, specificity: 0.973, PPV: 0.829, and NPV: 0.993. Dice scores for intraparenchymal hemorrhage: 0.931, epidural/subdural hemorrhage: 0.863. SAH: 0.772.	10,841	CNN
[55], 2019—2c, 2d	Location: International Aim: Segmentation and phenotyping of acute ischemic lesions on MRI. Variables: MRI data Strengths: Single-center cohort: 267; MRI-GENIE cohort (from 12 international centers from the Stroke Genetics Network): 3301 Limitations: Retrospective Findings: No algorithm-specific results reported. Automated and manual lesion volumes were statistically correlated. Notable facts: Deep learning algorithms trained on diverse data can be successfully used for segmentation of clinical diffusion-weighted MRI lesions.	3568	CNN
[48], 2019—2b, 2c	Location: China Aim: Detecting ICH and subtypes (cerebral, parenchymal, intraventricular, subdural, epidural, and subarachnoid) in NCCT. Variables: CT scan image slices data Strengths: Multi-institutional Limitations: Retrospective; prevalence of ICH (65%) was higher than that in a real clinical setting; limited number of cases in some subtypes (case/control ratio of 1:14); comparison was made with junior radiology trainees Findings: AUC (detecting ICH): 0.98; AUC (detecting subtype): 0.8.	2836	CNN-RNN
[56], 2019—2c, 3b	Location: China Aim: Predicting hematoma expansion in patients with spontaneous ICH. Variables: Fibrinogen level, sex, Glasgow Coma Score, time to initial CT scan, black hole sign, blend sign, satellite sign, midline shift, and baseline hematoma volume Strengths: Large sample size Limitations: Retrospective Findings: Sensitivity: 81.3%; specificity: 84.8%; accuracy of 83.3%; AUC: 0.89. Notable facts: Potential utility in institutions where CTA is limited.	1157	SVM
[29], 2019—1b, 1c	Location: Japan Aim: Detecting cerebral aneurysms at time-of-flight MR angiography. Variables: MRA image data Limitations: Retrospective; variable number of training samples per aneurysm location Findings: Sensitivity: 91–93% Notable facts: The model improved aneurysm detection by 4.8–13% compared with the initial reports.	748	DL (ResNet)
[44], 2019—2b, 2c	Location: USA Aim: Using an automated algorithm to detect intracranial LVO on CTA. Variables: CTA image data Limitations: Retrospective Findings: Sensitivity: 92–94%, NPV: 97–98%; specificity 0.76–0.81. Notable facts: RAPID CTA can be used in the emergent setting as a screening tool to alert radiologists.	477	RAPID CTA
[43], 2019—2b, 2c	Location: USA Aim: Identifying LVO and ischemic core volume in patients using CTA. Variables: CTA image data Strengths: Comparison with RAPID CTA Limitations: Retrospective; 338 patients excluded mainly due to imaging artifacts/quality Findings: AUC (LVO detection): 0.88; AUC (Ischemic core detection ≤ 30 mL): 0.88; AUC (Ischemic core detection ≤ 50 mL): 0.90; AUC (early time window): 0.90; AUC (late time window): 0.91. Notable facts: CTA has the required information for neuroimaging evaluation of endovascular therapy with potential to be automated by ML.	297	CNN (DeepSymNet)
[90], 2018—3b	Location: Denmark Aim: Use deep learning to identify and combine acute imaging features of ischemic stroke to predict lesion volume. Variables: MRI data Strengths: Comparing different CNNs Limitations: Retrospective; no control group; model is potentially biased toward infarct overestimation Findings: AUC: 0.88 ± 0.12. Notable facts: CNN improved prediction accuracy over current methods.	222	CNN
[54], 2017—2c	Location: USA Aim: Distinguishing between hyperacute ischemic lesions and their corresponding contralateral brain tissue in NCCT Variables: CT image data Limitations: Retrospective; used contralateral hemisphere as control possibly capturing old ischemic lesions. Findings: AUC: 0.82. Notable facts: Optimal texture features provided to distinguish between hyperacute ischemic lesions and their corresponding contralateral brain tissue in NCCT.	139	SVM, Decision trees, AdaBoost
[57], 2019—2c	Location: USA and Australia Aim: Predicting ischemic core on CT perfusion image. Variables: CT image data Strengths: Included patients who underwent back-to-back CT perfusion imaging and MRI Limitations: Retrospective; possibly overestimating the ischemic core volume (due to the dependency on the arbitrary subregion of the brain) Findings: AUC (ischemic core prediction): 0.85–0.87; sensitivity (ischemic core prediction): 0.90–0.91; specificity (ischemic core prediction): 0.62–0.65; maximal Dice coefficient: 0.48. Notable facts: ANN accurately integrates clinical and CT perfusion imaging data to predict ischemic core.	128	ANN

** Category definition: *Category 1*: Application of AI in pre-diagnosis modeling: primary prevention (1a: Risk Estimation, 1b: Clustering/patient profiling before the event, 1c: Care gap identification and personalized prevention, 1d: Personalized prevention). *Category 2*: Application of AI in diagnosis and acute-phase treatment (2a: EMS proper referral, 2b: Acute Diagnosis, 2c: Acute Imaging, 2d: Triaging and Acute Treatment). *Category 3*: Application of AI in post-diagnosis outcome prediction and secondary prevention (3a: Personalize Treatment, 3b: Outcome prediction/effect disposition). *Category 4*: Application of AI in rehabilitation (4a: Personalize Treatment, 4b: Outcome Prediction). Abbreviations: ANN: artificial neural network; ADB: AdaBoost classifier; AF: atrial fibrillation; AUC: area under the curve; BC: binary classification; CART: classification and regression tree; CCI: Charlson comorbidity index; CDS: clinical decision support; CT: computed tomography; CTA: computed tomography angiogram; CTP: computed tomography perfusion; DL: deep learning; DNN: deep neural network; DT: decision tree; DWI: diffusion weighted image; EHR: electronic health record; ET: endovascular thrombectomy; FCN: fully convolutional neural network; FLIRT: FMRIB’s Linear Image Registration Tool; GBM: gradient boosting machine; GBRT: gradient boosted regression tree; GLM: generalized linear model; GNB: Gaussian naïve Bayes; GRU: Gated Recurrent Unit; ICH: intracranial hemorrhage; KNNC: K-nearest neighbor classifier; LR: linear regression; LOS: length of hospital stay; LVO: large vessel occlusion; FCN: fully convolutional neural network; MRI: magnetic resonance imaging; mRS: modified Rankin Scale score; NB: Naïve Bayes; NIHSS: National Institutes of Health Stroke Scale; PCP: primary care provider; PLS: partial least squares; RF: random forest; ROC: receiver operating characteristic; SMR: stepwise multilinear regression; SVM: support vector machine; TIA: transient ischemic attack; XGB: extreme gradient boosting.

## 4. Other Applications of AI

### 4.1. Clinical Trials in the AI-Era

Patient selection for a clinical trial is a crucial process, and research has shown that predictive modeling in the selection of patients would increase the trials’ success rate [121]. The development of a drug takes about ten years and more than two billion dollars, and yet only a fraction of drugs are approved by the Food and Drug Administration (FDA) [122]. The application of in silico clinical trials to suggest better patient selection criteria [123,124] can increase the efficiency and speed of drug development. For instance, the use of AI in clinical trials can increase the efficacy of screening of drug candidates based on (a) analysis of calculated properties, (b) prediction models for therapeutic drug targets, and (c) identification of safety liabilities; all of which facilitate a reduction in the number of in vivo or in vitro assay requirements [125]. These efforts are also driven by innovative start-up companies to reduce the cost and improve the success rate of trials. 

### 4.2. AI at Physicians’ Fingertips—Implication and Future Directions

Once validated and proven effective and safe, the AI solutions have to be integrated into clinical workflow and demonstrated to be effective in improving outcomes. It is only then that we have leaped to provide evidence-based care in real-time using the promises of big data and AI. However, taking the advances in AI to the bedside is not trivial. First, novel AI solutions must be rigorously assessed. Certainly, the FDA approval for AI applications is laying the foundation for regulatory evolution to allow faster integration of AI-enabled technologies into healthcare. Many clinical trials are designed to evaluate the impact of technological advances (such as new imaging devices [126]) like the drug-design trials. Second, carefully designed CDSS need to be developed and implemented in the EHR that take the AI-powered tool to physician’s fingertips. To achieve these goals, the American Medical Informatics Association (AMIA) published a roadmap [127] in 2007 for taking action on CDSS and defined three main pillars: (a) high adoption and effective use, (b) best knowledge available when needed, and (c) continuous improvement of knowledge and CDSS methods. However, in general, physicians have relatively positive attitudes toward the idea of CDSS [128,129], even though there are many challenges, including low specificity [130,131], workflow interruptions [132,133,134], confusing interfaces [135,136], low confidence [137], awareness of the information [138], requirements of manual data entry [134,139], interference with physician autonomy [128,140], or lack of relevance [134] that limit the effective use and adoption of CDSS in many health care systems. “Alert Fatigue” can be caused by poorly designed and implemented CDSS [128,141,142,143]. The four principles for the design of CDSS interfaces (four A’s: All in one, At a glance, At hand, and Attention) [144] should also be followed. Based on the unified theory of acceptance and use of technology [145], user expectations need to be taken into consideration for technology to be accepted. In addition, several studies highlighted the importance of considering the end-user needs and expectations early in the development process [139,143,146]. Therefore, it is imperative to have CDSS end-users involved in the design and implementation. It is also essential to consult EHR engineers and information technologists to understand the possibilities, limitations, and hardware/software requirements to effectively utilize CDSS functionalities. Careful planning requires mapping current workflows to understand how clinical phases and tasks are completed and how these may be affected by the addition of CDSS. In some instances, CDSS may need to be customized to suit various processes. Many physicians remain hesitant to accept CDSSs, leading to suboptimal implementation [143]. Finally, despite federal investment to promote health information technology adoption, gaps remain in the use of CDSS among health systems [147], and we believe that lack of physician acceptance may be one of the main reasons. Thus, it is imperative for researchers across the translational spectrum to be involved in this AI revolution so that we can together reach the promises of precision health in a scalable and fair manner. 

### 4.3. Health Disparity and Implicit Bias

Although recent scrutiny of AI-based software has introduced concern about unintended effects of AI on social bias and inequity [148], there are opportunities to leverage technology to reduce health disparity, care gaps [149,150], and unwanted variations [151], as well as improving access. There are many examples of how technology is improving access to specialty care, especially in rural areas. However, AI-based studies have to be carefully designed with explicit frameworks and a balanced representation of participants to mitigate some of the undesirable biases. For instance, the use of deep transfer learning is effective in reducing healthcare disparities that are driven by data inequality [152]. The reader is referred to the work by Cirillo et al. [153] for a more detailed overview and some of the recommendations on how to improve the global health and disease landscape and decrease inequalities with the use of technology. 

There are also other challenges and opportunities when integrating AI tools in clinical workflow; namely, there are technological challenges, operational challenges, and ethical challenges [61]. These issues are tightly intertwined with implicit biases and health disparity. Larger centers with better access to robust infrastructure and a wide range of patient representation are better positioned to address implicit biases and address these challenges, leading to better integration of AI-assistive tools in the clinical workflow. However, as it is impossible—in practical terms—to find solutions to ensure the highest efficacy, efficiency, equity, and patient safety, it is important and necessary to define acceptable thresholds by working meticulously with regulatory institutions to guide the development of AI tools to ensure best practices and compliance.

## 5. Conclusions

To summarize, we have seen that the field of AI is omnipresent in both cardio and cerebrovascular fields, targeting different stages of patient management (Figure 2). However, in the cardiovascular field, studies have been larger, and there were more prospective and multi-center studies. In the field of cerebrovascular diseases, studies were mostly retrospectives from single centers and limited in patient representation and scale. By enhancing collaborative efforts, future cerebrovascular studies can expand follow-up periods to better understand the long-term outcomes in the patients. Both cardio- and cerebrovascular fields can also benefit from collaborative efforts to increase data diversity, patient representation, and integration of different data modalities, e.g., imaging biomarkers and genetic information.

Currently, the limitations in AI-based models are mostly centered on the lack of sufficient patient representation, balanced cohorts, and biases introduced by cohort definitions or selection of variables, as well as the exclusion of a certain group of patients. Machine learning models pick up biases from the training datasets; therefore, to reach new heights, it is of fundamental importance to increase patient representation and data density and improve data for downstream modeling [154,155]. Finally, in terms of methodologies, both fields are taking advantage of advances in machine learning frameworks and tools. Ultimately, the future of healthcare is an organic blend of technology, innovation, and human connection. It is not enough to provide faster, better care; we must leverage the technology to also ensure that the care is fair and not biased towards a group or sub-population. We must understand our limitations and use the technology to deliver an integrated solution that does not make the physicians fixed to the screen and the keyboard. The care also has to ensure physicians receive the tools they need to be better at what they do. Overall, there are few areas in which AI can be of great value in both cardio and cerebrovascular diseases: (1) disease diagnosis and patient monitoring, especially in high-impact fields; (2) incidental findings for preventive care by scanning through images and reports; (3) risk stratification for primary or secondary prevention; and (4) resource and workflow optimization by leveraging administrative data.

## Data Availability

Not applicable.
